# 9-[(*E*)-2-(2-Meth­oxy­phen­yl)ethen­yl]-3,4,5,6,7,9-hexa­hydro-2*H*-xanthene-1,8-dione

**DOI:** 10.1107/S1600536812001419

**Published:** 2012-01-18

**Authors:** Joo Hwan Cha, Ae Nim Pae, Jae Kyun Lee, Yong Seo Cho

**Affiliations:** a Advanced Analysis Center, Korea Institute of Science & Technology, Hwarangro 14-gil, Seongbuk-gu, Seoul 136-791, Republic of Korea; b Center for Neuro-Medicine, Korea Institute of Science & Technology, Hwarangro 14-gil, Seongbuk-gu, Seoul 136-791, Republic of Korea

## Abstract

In the title compound, C_22_H_22_O_4_, the two cyclo­hexenone rings adopt half-chair conformations, whereas the six-membered pyran ring adopts a flattened boat conformation, with the O and methine C atoms deviating from the plane of the other four atoms by 0.142 (2) and 0.287 (2)Å, respectively. In the crystal, weak C—H⋯O hydrogen bonds link mol­ecules into chains running parallel to the *a* axis.

## Related literature

For the biological activity of xanthenes and their derivatives, see: Lee *et al.* (2011 ▶). For related structures of xanthenes, see: Asad *et al.* (2012 ▶); Fun *et al.* (2011 ▶); Mehdi *et al.* (2011 ▶).
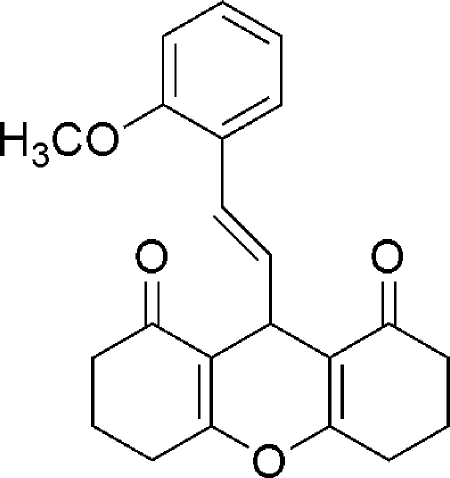


## Experimental

### 

#### Crystal data

C_22_H_22_O_4_
*M* *_r_* = 350.41
Monoclinic, 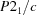

*a* = 8.5396 (5) Å
*b* = 9.9243 (7) Å
*c* = 21.9501 (13) Å
β = 102.5455 (14)°
*V* = 1815.85 (19) Å^3^
*Z* = 4
Mo *K*α radiation
μ = 0.09 mm^−1^
*T* = 296 K
0.40 × 0.20 × 0.20 mm

#### Data collection

Rigaku R-AXIS RAPID diffractometer
Absorption correction: multi-scan (*ABSCOR*; Rigaku, 1995 ▶) *T*_min_ = 0.821, *T*_max_ = 0.983
17353 measured reflections
4100 independent reflections
3248 reflections with *F* ^2^ > 2σ(*F* ^2^)
*R*_int_ = 0.018

#### Refinement

*R*[*F* ^2^ > 2σ(*F* ^2^)] = 0.044
*wR*(*F* ^2^) = 0.136
*S* = 1.08
4100 reflections
244 parameters
H atoms treated by a mixture of independent and constrained refinement
Δρ_max_ = 0.25 e Å^−3^
Δρ_min_ = −0.21 e Å^−3^

### 

Data collection: *RAPID-AUTO* (Rigaku, 2006 ▶); cell refinement: *RAPID-AUTO*; data reduction: *RAPID-AUTO*; program(s) used to solve structure: *SHELXS97* (Sheldrick, 2008 ▶); program(s) used to refine structure: *SHELXL97* (Sheldrick, 2008 ▶); molecular graphics: *CrystalStructure* (Rigaku, 2010 ▶); software used to prepare material for publication: *CrystalStructure*.

## Supplementary Material

Crystal structure: contains datablock(s) global, I. DOI: 10.1107/S1600536812001419/go2043sup1.cif

Structure factors: contains datablock(s) I. DOI: 10.1107/S1600536812001419/go2043Isup2.hkl

Supplementary material file. DOI: 10.1107/S1600536812001419/go2043Isup3.cml

Additional supplementary materials:  crystallographic information; 3D view; checkCIF report

## Figures and Tables

**Table 1:** Hydrogen-bond geometry (Å, °)

*D*—H⋯*A*	*D*—H	H⋯*A*	*D*⋯*A*	*D*—H⋯*A*
C14—H14*B*⋯O2^i^	0.97	2.43	3.3231 (18)	153

Symmetry code: (i) 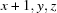.

**Fig. 1.**
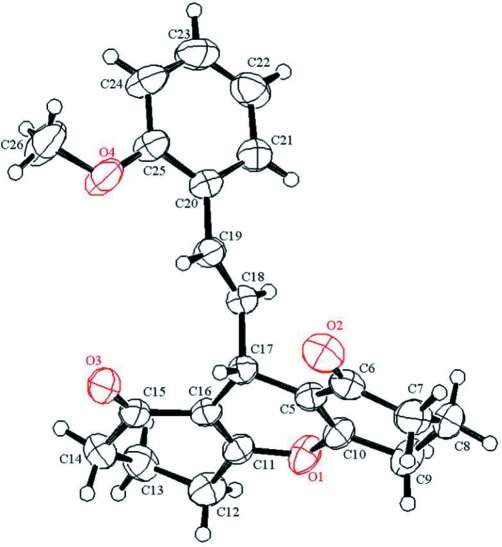
The molecular structure of (I) showing the atomic numbering and 50% probability displacement ellipsoid.

## supplementary crystallographic information

### Comment

As part of our ongoing study of the substituent effect on the solid state structures of xanthene derivatives (Lee *et al.*, 2011).

In C_22_H_22_O_4_, (Fig. 1), the bond lengths and angles are normal and correspond to those observed in related structures (Asad *et al.*, 2012; Fun *et al.*, 2011; Mehdi *et al.*, 2011). The methoxyphenyl group in molecule is almost planar [C26–O4–C25–C26 = -174.54 (16)°]. The two cyclohexenone rings in display half-chair conformation, Fig. 1. Atom C4 lies 0.621 (2)Å above the plane of the remaining 5 atoms of the ring containing atoms C1 to C6. Atom C9 lies 0.621 (2)Å above the plane of the remaining 5 atoms of the ring containing atoms C7 to C12. In the six-membered xanthene ring adopts a flattened boat conformation with the O1 and methine, 13, atoms deviating from the plane of the other four atoms by 0.142 (2)Å and 0.287 (2) Å.

In the crystal, a weak intermolecular C—H···O hydrogen bonds (Table 1) links the molecules into chains which run parallel to the *a*-axis.

### Experimental

To a solution of 3-Hydroxy-2-[(2(*E*))-1-(2-hydroxy-6-oxocyclohex-1-en-1-yl)-3- (2-methoxyphenyl)prop-2-en-1-yl]cyclohex-2-en-1-one (1.25 mmol) was added methanol(12.5 mL) and catalytic amounts of sulfuric acid(0.2 mL) under a nitrogen atmosphere. After stirring for 3 h, the solvent was evaporated and the remaining residue dissolved in ethyl acetate. The mixture was neutralized with saturated sodium bicarbonate and the solution was extracted with ethyl acetate. The resulting solid residue was purified by recrystallization from ethanol and methylene chloride to afford white needle crystals suitable for X-ray analysis.

### Refinement

Atoms H18 and H19 were located from a difference Fourier map and refined freely [C18—H = 0.965 (18) Å and C19—H = 1.00 (2) Å]. The remaining hydrogen atoms were positioned geometrically and refined using a riding model with [C—H = 0.93–0.97 Å] and *U*iso(H) = 1.2 or 1.5*U*eq(C).

### Crystal data

**Table:**

C_22_H_22_O_4_	*F*(000) = 744.00
*M**_r_* = 350.41	*D*_x_ = 1.282 Mg m^−^^3^
Monoclinic, *P*2_1_/*c*	Melting point: 427 K
Hall symbol: -P 2ybc	Mo *K*α radiation, λ = 0.71075 Å
*a* = 8.5396 (5) Å	Cell parameters from 13636 reflections
*b* = 9.9243 (7) Å	θ = 3.2–27.4°
*c* = 21.9501 (13) Å	µ = 0.09 mm^−^^1^
β = 102.5455 (14)°	*T* = 296 K
*V* = 1815.85 (19) Å^3^	Block, colourless
*Z* = 4	0.40 × 0.20 × 0.20 mm

### Data collection

**Table:**

Rigaku R-AXIS RAPID diffractometer	3248 reflections with *F*^2^ > 2σ(*F*^2^)
Detector resolution: 10.000 pixels mm^-1^	*R*_int_ = 0.018
ω scans	θ_max_ = 27.4°
Absorption correction: multi-scan (*ABSCOR*; Rigaku, 1995)	*h* = −10→11
*T*_min_ = 0.821, *T*_max_ = 0.983	*k* = −12→12
17353 measured reflections	*l* = −28→26
4100 independent reflections	

### Refinement

**Table:**

Refinement on *F*^2^	Secondary atom site location: difference Fourier map
*R*[*F*^2^ > 2σ(*F*^2^)] = 0.044	Hydrogen site location: inferred from neighbouring sites
*wR*(*F*^2^) = 0.136	H atoms treated by a mixture of independent and constrained refinement
*S* = 1.08	*w* = 1/[σ^2^(*F*_o_^2^) + (0.0784*P*)^2^ + 0.1912*P*] where *P* = (*F*_o_^2^ + 2*F*_c_^2^)/3
4100 reflections	(Δ/σ)_max_ < 0.001
244 parameters	Δρ_max_ = 0.25 e Å^−^^3^
0 restraints	Δρ_min_ = −0.21 e Å^−^^3^
Primary atom site location: structure-invariant direct methods	

### Special details

**Table:**

Geometry. All e.s.d.'s (except the e.s.d. in the dihedral angle between two l.s. planes) are estimated using the full covariance matrix. The cell e.s.d.'s are taken into account individually in the estimation of e.s.d.'s in distances, angles and torsion angles; correlations between e.s.d.'s in cell parameters are only used when they are defined by crystal symmetry. An approximate (isotropic) treatment of cell e.s.d.'s is used for estimating e.s.d.'s involving l.s. planes.
Refinement. Refinement was performed using all reflections. The weighted *R*-factor (*wR*) and goodness of fit (*S*) are based on *F*^2^. *R*-factor (gt) are based on *F*. The threshold expression of *F*^2^ > 2.0 σ(*F*^2^) is used only for calculating *R*-factor (gt).

### Fractional atomic coordinates and isotropic or equivalent isotropic displacement parameters (Å^2^)

**Table:**

	*x*	*y*	*z*	*U*_iso_*/*U*_eq_	
O1	0.24674 (12)	0.65221 (10)	0.34715 (4)	0.0507 (3)	
O2	−0.20315 (12)	0.36602 (11)	0.29337 (6)	0.0626 (3)	
O3	0.23752 (13)	0.39194 (11)	0.16669 (5)	0.0577 (3)	
O4	−0.19930 (17)	0.46300 (12)	0.02525 (5)	0.0704 (4)	
C5	0.01484 (14)	0.51591 (12)	0.31309 (6)	0.0390 (3)	
C6	−0.11815 (15)	0.44136 (14)	0.33047 (6)	0.0454 (3)	
C7	−0.13815 (18)	0.45708 (18)	0.39689 (7)	0.0592 (4)	
C8	−0.09477 (19)	0.59700 (18)	0.42221 (7)	0.0599 (4)	
C9	0.07687 (19)	0.63093 (17)	0.41853 (6)	0.0556 (4)	
C10	0.10633 (15)	0.59497 (13)	0.35617 (6)	0.0423 (3)	
C11	0.30878 (15)	0.60472 (13)	0.29859 (5)	0.0404 (3)	
C12	0.47915 (17)	0.64695 (16)	0.30453 (7)	0.0530 (4)	
C13	0.52343 (17)	0.64744 (16)	0.24128 (8)	0.0552 (4)	
C14	0.47594 (18)	0.51597 (17)	0.20733 (8)	0.0563 (4)	
C15	0.30516 (16)	0.47312 (13)	0.20547 (6)	0.0443 (3)	
C16	0.22483 (14)	0.52783 (12)	0.25284 (5)	0.0381 (3)	
C17	0.04913 (14)	0.49631 (12)	0.24894 (5)	0.0378 (3)	
C18	−0.05446 (14)	0.58600 (13)	0.20051 (6)	0.0395 (3)	
C19	−0.14029 (15)	0.54299 (14)	0.14667 (6)	0.0428 (3)	
C20	−0.23750 (15)	0.62750 (13)	0.09714 (6)	0.0423 (3)	
C21	−0.30228 (17)	0.75056 (15)	0.10949 (7)	0.0511 (4)	
C22	−0.3946 (2)	0.82777 (17)	0.06197 (8)	0.0637 (5)	
C23	−0.4239 (3)	0.78154 (19)	0.00164 (8)	0.0697 (5)	
C24	−0.3609 (2)	0.65984 (18)	−0.01266 (7)	0.0656 (5)	
C25	−0.26841 (18)	0.58327 (15)	0.03475 (6)	0.0504 (4)	
C26	−0.2132 (4)	0.4176 (3)	−0.03727 (8)	0.0919 (8)	
H7A	−0.2487	0.4381	0.3984	0.0711*	
H7B	−0.0706	0.3918	0.4232	0.0711*	
H8A	−0.1054	0.6018	0.4653	0.0719*	
H8B	−0.1677	0.6622	0.3982	0.0719*	
H9A	0.0958	0.7265	0.4260	0.0667*	
H9B	0.1510	0.5819	0.4507	0.0667*	
H12A	0.5491	0.5855	0.3322	0.0636*	
H12B	0.4943	0.7365	0.3226	0.0636*	
H13A	0.4694	0.7216	0.2165	0.0662*	
H13B	0.6382	0.6608	0.2467	0.0662*	
H14A	0.4902	0.5246	0.1649	0.0676*	
H14B	0.5477	0.4456	0.2275	0.0676*	
H17	0.0289	0.4020	0.2365	0.0453*	
H21	−0.2835	0.7819	0.1504	0.0613*	
H22	−0.4362	0.9101	0.0711	0.0765*	
H23	−0.4869	0.8325	−0.0300	0.0836*	
H24	−0.3804	0.6297	−0.0537	0.0787*	
H26A	−0.1731	0.4857	−0.0609	0.1102*	
H26B	−0.3239	0.4000	−0.0558	0.1102*	
H26C	−0.1520	0.3364	−0.0372	0.1102*	
H18	−0.0539 (19)	0.6800 (18)	0.2117 (8)	0.056 (5)*	
H19	−0.138 (2)	0.444 (2)	0.1371 (8)	0.063 (5)*	

### Atomic displacement parameters (Å^2^)

**Table:**

	*U*^11^	*U*^22^	*U*^33^	*U*^12^	*U*^13^	*U*^23^
O1	0.0564 (6)	0.0566 (6)	0.0404 (5)	−0.0216 (5)	0.0137 (4)	−0.0100 (4)
O2	0.0491 (6)	0.0578 (7)	0.0788 (8)	−0.0151 (5)	0.0095 (6)	−0.0031 (6)
O3	0.0601 (6)	0.0613 (7)	0.0500 (6)	0.0067 (5)	0.0082 (5)	−0.0137 (5)
O4	0.1119 (10)	0.0573 (7)	0.0361 (6)	0.0187 (7)	0.0031 (6)	−0.0024 (5)
C5	0.0399 (6)	0.0381 (7)	0.0375 (6)	−0.0010 (5)	0.0052 (5)	0.0046 (5)
C6	0.0379 (7)	0.0420 (7)	0.0549 (8)	0.0011 (5)	0.0073 (6)	0.0097 (6)
C7	0.0505 (8)	0.0719 (11)	0.0580 (9)	−0.0028 (7)	0.0179 (7)	0.0191 (8)
C8	0.0641 (9)	0.0766 (11)	0.0430 (8)	0.0088 (8)	0.0204 (7)	0.0077 (7)
C9	0.0664 (10)	0.0630 (10)	0.0388 (7)	−0.0083 (7)	0.0145 (7)	−0.0032 (6)
C10	0.0483 (7)	0.0430 (7)	0.0359 (6)	−0.0056 (5)	0.0095 (5)	0.0030 (5)
C11	0.0443 (7)	0.0407 (7)	0.0355 (6)	−0.0055 (5)	0.0074 (5)	0.0041 (5)
C12	0.0464 (8)	0.0569 (9)	0.0533 (8)	−0.0141 (6)	0.0055 (6)	0.0024 (7)
C13	0.0456 (8)	0.0576 (9)	0.0658 (9)	−0.0008 (6)	0.0198 (7)	0.0071 (7)
C14	0.0485 (8)	0.0620 (10)	0.0607 (9)	0.0086 (7)	0.0168 (7)	0.0000 (7)
C15	0.0485 (7)	0.0427 (7)	0.0402 (7)	0.0091 (6)	0.0062 (6)	0.0033 (6)
C16	0.0410 (6)	0.0363 (7)	0.0353 (6)	0.0004 (5)	0.0045 (5)	0.0043 (5)
C17	0.0412 (7)	0.0338 (6)	0.0360 (6)	−0.0027 (5)	0.0031 (5)	−0.0013 (5)
C18	0.0407 (7)	0.0373 (7)	0.0388 (6)	−0.0019 (5)	0.0052 (5)	0.0012 (5)
C19	0.0479 (7)	0.0412 (7)	0.0369 (7)	0.0015 (6)	0.0042 (6)	0.0001 (5)
C20	0.0438 (7)	0.0432 (7)	0.0377 (6)	−0.0031 (5)	0.0040 (5)	0.0040 (5)
C21	0.0532 (8)	0.0506 (8)	0.0489 (8)	0.0032 (6)	0.0098 (6)	0.0034 (6)
C22	0.0654 (10)	0.0534 (9)	0.0720 (11)	0.0142 (8)	0.0144 (8)	0.0146 (8)
C23	0.0723 (11)	0.0703 (11)	0.0604 (10)	0.0116 (9)	0.0013 (8)	0.0249 (8)
C24	0.0814 (12)	0.0664 (11)	0.0415 (8)	0.0013 (9)	−0.0033 (7)	0.0109 (7)
C25	0.0604 (9)	0.0479 (8)	0.0391 (7)	−0.0010 (6)	0.0029 (6)	0.0043 (6)
C26	0.159 (3)	0.0715 (13)	0.0406 (9)	0.0177 (13)	0.0119 (11)	−0.0068 (8)

### Geometric parameters (Å, °)

**Table:**

O1—C10	1.3794 (17)	C22—C23	1.372 (3)
O1—C11	1.3727 (17)	C23—C24	1.386 (3)
O2—C6	1.2216 (17)	C24—C25	1.388 (2)
O3—C15	1.2222 (17)	C7—H7A	0.970
O4—C25	1.367 (2)	C7—H7B	0.970
O4—C26	1.425 (3)	C8—H8A	0.970
C5—C6	1.4734 (19)	C8—H8B	0.970
C5—C10	1.3414 (17)	C9—H9A	0.970
C5—C17	1.5118 (19)	C9—H9B	0.970
C6—C7	1.512 (3)	C12—H12A	0.970
C7—C8	1.512 (3)	C12—H12B	0.970
C8—C9	1.523 (3)	C13—H13A	0.970
C9—C10	1.488 (2)	C13—H13B	0.970
C11—C12	1.492 (2)	C14—H14A	0.970
C11—C16	1.3386 (16)	C14—H14B	0.970
C12—C13	1.516 (3)	C17—H17	0.980
C13—C14	1.514 (3)	C18—H18	0.965 (18)
C14—C15	1.511 (2)	C19—H19	1.00 (2)
C15—C16	1.4685 (19)	C21—H21	0.930
C16—C17	1.5168 (17)	C22—H22	0.930
C17—C18	1.5154 (16)	C23—H23	0.930
C18—C19	1.3190 (17)	C24—H24	0.930
C19—C20	1.4773 (18)	C26—H26A	0.960
C20—C21	1.391 (2)	C26—H26B	0.960
C20—C25	1.4074 (19)	C26—H26C	0.960
C21—C22	1.393 (3)		
			
O1···C17	2.8824 (14)	C16···H21^i^	3.2029
O2···C10	3.5306 (16)	C17···H21^i^	3.3906
O2···C17	2.8596 (18)	C17···H18^i^	3.254 (18)
O2···C18	3.4143 (19)	C18···H13B^ii^	3.1026
O3···C11	3.5280 (16)	C18···H17^v^	3.4151
O3···C17	2.8605 (18)	C19···H9A^i^	3.5797
O3···C18	3.3588 (18)	C19···H13B^ii^	3.4021
O3···C19	3.4977 (17)	C19···H14A^ii^	3.2706
O4···C19	2.7210 (17)	C20···H14A^ii^	3.1875
C5···C8	2.870 (3)	C21···H13A^ii^	3.3745
C5···C11	2.7434 (19)	C21···H13B^ii^	3.2853
C6···C9	2.941 (2)	C21···H14A^ii^	3.2528
C6···C18	3.342 (2)	C21···H26B^xi^	3.4900
C7···C10	2.800 (3)	C22···H7A^xii^	3.5191
C10···C16	2.7585 (19)	C22···H9B^v^	3.3189
C10···C18	3.3955 (18)	C22···H23^xiii^	3.5464
C11···C14	2.840 (3)	C22···H26B^xi^	3.2808
C11···C18	3.3749 (16)	C23···H8A^xiv^	3.2128
C12···C15	2.9169 (19)	C23···H8B^xiv^	3.5230
C13···C16	2.873 (2)	C23···H22^xiii^	3.5367
C15···C18	3.2485 (19)	C23···H26B^xi^	3.2259
C16···C19	3.4668 (16)	C24···H8A^xiv^	3.3244
C18···C21	3.0497 (18)	C24···H8B^xiv^	3.3273
C20···C23	2.796 (2)	C24···H26B^xi^	3.4078
C21···C24	2.769 (3)	C26···H7B^xv^	3.4816
C22···C25	2.772 (3)	C26···H9A^i^	3.0831
C24···C26	2.821 (3)	C26···H14A^iv^	3.2999
O2···C11^i^	3.2897 (17)	H7A···C12^ii^	3.4446
O2···C12^i^	3.5636 (18)	H7A···C22^xvi^	3.5191
O2···C13^i^	3.4440 (18)	H7A···H9B^ix^	3.2417
O2···C14^ii^	3.3231 (18)	H7A···H12A^ii^	2.4784
O2···C16^i^	3.4995 (17)	H7A···H13A^i^	3.5313
O2···C18^i^	3.5287 (17)	H7A···H22^xvi^	2.9242
O3···C8^i^	3.582 (2)	H7A···H26C^x^	3.0971
O3···C12^iii^	3.3913 (19)	H7B···C8^ix^	3.3822
O3···C13^iii^	3.5135 (19)	H7B···C9^ix^	3.4933
O3···C26^iv^	3.381 (3)	H7B···C26^x^	3.4816
O4···C9^i^	3.594 (2)	H7B···H8A^ix^	2.5805
C8···O3^v^	3.582 (2)	H7B···H9A^ix^	3.5632
C9···O4^v^	3.594 (2)	H7B···H9B^ix^	3.0054
C11···O2^v^	3.2897 (17)	H7B···H26C^x^	2.5748
C12···O2^v^	3.5636 (18)	H8A···C7^ix^	3.3300
C12···O3^vi^	3.3913 (19)	H8A···C8^ix^	3.3287
C13···O2^v^	3.4440 (18)	H8A···C9^ix^	3.4102
C13···O3^vi^	3.5135 (19)	H8A···C23^xvii^	3.2128
C14···O2^vii^	3.3231 (18)	H8A···C24^xvii^	3.3244
C16···O2^v^	3.4995 (17)	H8A···H7B^ix^	2.5805
C18···O2^v^	3.5287 (17)	H8A···H8A^ix^	2.9077
C26···O3^iv^	3.381 (3)	H8A···H9B^ix^	2.6817
O1···H9A	2.4835	H8A···H23^xvii^	3.3459
O1···H9B	2.6691	H8A···H24^xvii^	3.5166
O1···H12A	2.7531	H8A···H26C^v^	3.3528
O1···H12B	2.4400	H8B···O3^v^	2.6864
O1···H18	3.491 (14)	H8B···C12^ii^	3.2638
O2···H7A	2.5240	H8B···C23^xvii^	3.5230
O2···H7B	2.8402	H8B···C24^xvii^	3.3273
O2···H17	2.5864	H8B···H12A^ii^	2.6466
O3···H14A	2.5356	H8B···H12B^ii^	3.0898
O3···H14B	2.7472	H8B···H13B^ii^	3.3780
O3···H17	2.5913	H8B···H23^xvii^	3.4318
O3···H19	3.176 (17)	H8B···H24^xvii^	3.0840
O4···H24	2.6398	H9A···O3^v^	3.5237
O4···H19	2.403 (17)	H9A···O4^v^	2.6506
C5···H7A	3.3165	H9A···C19^v^	3.5797
C5···H7B	2.9423	H9A···C26^v^	3.0831
C5···H8B	3.0486	H9A···H7B^ix^	3.5632
C5···H9A	3.2000	H9A···H23^viii^	3.5333
C5···H9B	3.0614	H9A···H26B^v^	3.5306
C5···H18	2.716 (17)	H9A···H26C^v^	2.6222
C6···H8A	3.3407	H9A···H19^v^	2.6340
C6···H8B	2.7324	H9B···C7^ix^	3.3941
C6···H9B	3.3988	H9B···C8^ix^	3.4281
C6···H17	2.6669	H9B···C22^i^	3.3189
C7···H9A	3.3156	H9B···H7A^ix^	3.2417
C7···H9B	2.7851	H9B···H7B^ix^	3.0054
C9···H7A	3.3243	H9B···H8A^ix^	2.6817
C9···H7B	2.6989	H9B···H22^i^	3.0932
C10···H7B	3.0788	H9B···H23^viii^	3.1459
C10···H8A	3.2998	H9B···H26C^v^	3.1600
C10···H8B	2.7770	H12A···O2^vii^	3.2759
C10···H17	3.2020	H12A···O3^vi^	3.5430
C10···H18	3.279 (15)	H12A···C6^vii^	3.1890
C11···H13A	2.7476	H12A···C7^vii^	3.0207
C11···H13B	3.3028	H12A···C8^vii^	3.2487
C11···H14B	3.2370	H12A···H7A^vii^	2.4784
C11···H17	3.1942	H12A···H8B^vii^	2.6466
C11···H18	3.350 (15)	H12A···H22^i^	3.0580
C12···H14A	3.3179	H12A···H23^viii^	3.2099
C12···H14B	2.7616	H12B···O2^v^	3.4010
C14···H12A	2.7637	H12B···O3^vi^	2.7289
C14···H12B	3.3231	H12B···C14^vi^	2.8748
C15···H12A	3.2873	H12B···C15^vi^	3.0475
C15···H13A	2.8211	H12B···H8B^vii^	3.0898
C15···H13B	3.3550	H12B···H14A^vi^	2.8735
C15···H17	2.6877	H12B···H14B^vi^	2.3401
C16···H12A	2.9892	H12B···H23^viii^	3.2759
C16···H12B	3.2244	H12B···H24^viii^	3.0026
C16···H13A	3.0694	H13A···O2^v^	2.6570
C16···H14A	3.2814	H13A···O3^vi^	3.5930
C16···H14B	3.0409	H13A···C14^vi^	3.3485
C16···H18	2.798 (16)	H13A···C15^vi^	3.3823
C17···H19	2.674 (16)	H13A···C21^vii^	3.3745
C18···H21	2.8076	H13A···H7A^v^	3.5313
C19···H17	2.5875	H13A···H14B^vi^	2.5611
C19···H21	2.6773	H13A···H21^vii^	2.8718
C20···H22	3.2638	H13B···O2^vii^	3.2921
C20···H24	3.2712	H13B···O3^vi^	3.0209
C20···H18	2.710 (15)	H13B···C5^vii^	3.5354
C21···H23	3.2289	H13B···C6^vii^	3.2845
C21···H18	2.821 (15)	H13B···C15^vi^	3.2748
C21···H19	3.346 (19)	H13B···C18^vii^	3.1026
C22···H24	3.2347	H13B···C19^vii^	3.4021
C23···H21	3.2240	H13B···C21^vii^	3.2853
C24···H22	3.2365	H13B···H8B^vii^	3.3780
C24···H26A	2.7240	H13B···H14B^vi^	3.3504
C24···H26B	2.7887	H13B···H21^vii^	2.6425
C25···H21	3.2381	H13B···H18^vii^	2.9010
C25···H23	3.2351	H14A···C19^vii^	3.2706
C25···H26A	2.5989	H14A···C20^vii^	3.1875
C25···H26B	2.6594	H14A···C21^vii^	3.2528
C25···H26C	3.1889	H14A···C26^iv^	3.2999
C25···H19	2.664 (17)	H14A···H12B^iii^	2.8735
C26···H24	2.5253	H14A···H21^vii^	3.2594
H7A···H8A	2.3466	H14A···H24^iv^	2.8601
H7A···H8B	2.3287	H14A···H26A^iv^	3.1406
H7B···H8A	2.3240	H14A···H26B^iv^	2.6140
H7B···H8B	2.8264	H14A···H19^vii^	3.4518
H7B···H9B	2.6469	H14B···O2^vii^	2.4300
H8A···H9A	2.4231	H14B···C6^vii^	3.2328
H8A···H9B	2.2893	H14B···C12^iii^	3.0435
H8B···H9A	2.2903	H14B···C13^iii^	3.1272
H8B···H9B	2.8295	H14B···H12B^iii^	2.3401
H12A···H13A	2.8269	H14B···H13A^iii^	2.5611
H12A···H13B	2.2977	H14B···H13B^iii^	3.3504
H12A···H14B	2.6837	H17···C18^i^	3.4151
H12B···H13A	2.2976	H17···H21^i^	3.1574
H12B···H13B	2.3951	H17···H18^i^	2.4669
H13A···H14A	2.2853	H21···C5^v^	3.2403
H13A···H14B	2.8167	H21···C10^v^	3.4721
H13B···H14A	2.3803	H21···C11^v^	3.4161
H13B···H14B	2.2784	H21···C13^ii^	3.1484
H17···H18	2.8706	H21···C16^v^	3.2029
H17···H19	2.3715	H21···C17^v^	3.3906
H21···H22	2.3157	H21···H13A^ii^	2.8718
H21···H18	2.3526	H21···H13B^ii^	2.6425
H22···H23	2.2996	H21···H14A^ii^	3.2594
H23···H24	2.3138	H21···H17^v^	3.1574
H24···H26A	2.3072	H22···O1^v^	3.2171
H24···H26B	2.3327	H22···C10^v^	3.4490
H24···H26C	3.4785	H22···C11^v^	3.4272
H18···H19	2.86 (3)	H22···C23^xiii^	3.5367
O1···H22^i^	3.2171	H22···H7A^xii^	2.9242
O1···H23^viii^	3.1309	H22···H9B^v^	3.0932
O1···H19^v^	3.086 (19)	H22···H12A^v^	3.0580
O2···H12A^ii^	3.2759	H22···H22^xiii^	3.5533
O2···H12B^i^	3.4010	H22···H23^xiii^	2.7407
O2···H13A^i^	2.6570	H23···O1^xviii^	3.1309
O2···H13B^ii^	3.2921	H23···C12^xviii^	3.5857
O2···H14B^ii^	2.4300	H23···C22^xiii^	3.5464
O2···H18^i^	2.889 (17)	H23···H8A^xiv^	3.3459
O3···H8B^i^	2.6864	H23···H8B^xiv^	3.4318
O3···H9A^i^	3.5237	H23···H9A^xviii^	3.5333
O3···H12A^iii^	3.5430	H23···H9B^xviii^	3.1459
O3···H12B^iii^	2.7289	H23···H12A^xviii^	3.2099
O3···H13A^iii^	3.5930	H23···H12B^xviii^	3.2759
O3···H13B^iii^	3.0209	H23···H22^xiii^	2.7407
O3···H24^iv^	2.9995	H23···H26B^xi^	3.5780
O3···H26A^iv^	2.5713	H24···O3^iv^	2.9995
O3···H26B^iv^	3.3935	H24···C14^iv^	3.5955
O4···H9A^i^	2.6506	H24···H8A^xiv^	3.5166
O4···H26A^iv^	3.1469	H24···H8B^xiv^	3.0840
O4···H26C^iv^	3.5623	H24···H12B^xviii^	3.0026
C5···H13B^ii^	3.5354	H24···H14A^iv^	2.8601
C5···H21^i^	3.2403	H26A···O3^iv^	2.5713
C5···H18^i^	3.406 (18)	H26A···O4^iv^	3.1469
C6···H12A^ii^	3.1890	H26A···C15^iv^	3.1544
C6···H13B^ii^	3.2845	H26A···H14A^iv^	3.1406
C6···H14B^ii^	3.2328	H26A···H26A^iv^	3.5410
C6···H18^i^	3.215 (18)	H26A···H26C^iv^	3.5872
C7···H8A^ix^	3.3300	H26A···H19^iv^	3.5032
C7···H9B^ix^	3.3941	H26B···O3^iv^	3.3935
C7···H12A^ii^	3.0207	H26B···C14^iv^	3.4019
C7···H26C^x^	3.2654	H26B···C15^iv^	3.5549
C8···H7B^ix^	3.3822	H26B···C21^xi^	3.4900
C8···H8A^ix^	3.3287	H26B···C22^xi^	3.2808
C8···H9B^ix^	3.4281	H26B···C23^xi^	3.2259
C8···H12A^ii^	3.2487	H26B···C24^xi^	3.4078
C9···H7B^ix^	3.4933	H26B···H9A^i^	3.5306
C9···H8A^ix^	3.4102	H26B···H14A^iv^	2.6140
C9···H26C^v^	3.2607	H26B···H23^xi^	3.5780
C9···H19^v^	3.423 (19)	H26C···O4^iv^	3.5623
C10···H21^i^	3.4721	H26C···C7^xv^	3.2654
C10···H22^i^	3.4490	H26C···C9^i^	3.2607
C10···H19^v^	3.48 (2)	H26C···H7A^xv^	3.0971
C11···H21^i^	3.4161	H26C···H7B^xv^	2.5748
C11···H22^i^	3.4272	H26C···H8A^i^	3.3528
C12···H7A^vii^	3.4446	H26C···H9A^i^	2.6222
C12···H8B^vii^	3.2638	H26C···H9B^i^	3.1600
C12···H14B^vi^	3.0435	H26C···H26A^iv^	3.5872
C12···H23^viii^	3.5857	H18···O2^v^	2.889 (17)
C13···H14B^vi^	3.1272	H18···C5^v^	3.406 (18)
C13···H21^vii^	3.1484	H18···C6^v^	3.215 (18)
C14···H12B^iii^	2.8748	H18···C17^v^	3.254 (18)
C14···H13A^iii^	3.3485	H18···H13B^ii^	2.9010
C14···H24^iv^	3.5955	H18···H17^v^	2.4669
C14···H26B^iv^	3.4019	H19···O1^i^	3.086 (19)
C15···H12B^iii^	3.0475	H19···C9^i^	3.423 (19)
C15···H13A^iii^	3.3823	H19···C10^i^	3.48 (2)
C15···H13B^iii^	3.2748	H19···H9A^i^	2.6340
C15···H26A^iv^	3.1544	H19···H14A^ii^	3.4518
C15···H26B^iv^	3.5549	H19···H26A^iv^	3.5032
			
C10—O1—C11	117.66 (10)	C7—C8—H8A	109.616
C25—O4—C26	118.34 (13)	C7—C8—H8B	109.611
C6—C5—C10	118.35 (13)	C9—C8—H8A	109.619
C6—C5—C17	119.52 (11)	C9—C8—H8B	109.615
C10—C5—C17	122.06 (12)	H8A—C8—H8B	108.140
O2—C6—C5	120.97 (13)	C8—C9—H9A	109.512
O2—C6—C7	122.05 (14)	C8—C9—H9B	109.511
C5—C6—C7	116.91 (12)	C10—C9—H9A	109.516
C6—C7—C8	112.29 (14)	C10—C9—H9B	109.524
C7—C8—C9	110.21 (14)	H9A—C9—H9B	108.079
C8—C9—C10	110.65 (12)	C11—C12—H12A	109.510
O1—C10—C5	122.08 (13)	C11—C12—H12B	109.508
O1—C10—C9	111.05 (11)	C13—C12—H12A	109.507
C5—C10—C9	126.86 (14)	C13—C12—H12B	109.512
O1—C11—C12	111.66 (11)	H12A—C12—H12B	108.072
O1—C11—C16	122.96 (12)	C12—C13—H13A	109.490
C12—C11—C16	125.36 (13)	C12—C13—H13B	109.492
C11—C12—C13	110.69 (11)	C14—C13—H13A	109.484
C12—C13—C14	110.77 (14)	C14—C13—H13B	109.490
C13—C14—C15	114.19 (14)	H13A—C13—H13B	108.066
O3—C15—C14	120.77 (14)	C13—C14—H14A	108.709
O3—C15—C16	120.94 (13)	C13—C14—H14B	108.702
C14—C15—C16	118.25 (12)	C15—C14—H14A	108.712
C11—C16—C15	119.05 (12)	C15—C14—H14B	108.705
C11—C16—C17	121.46 (12)	H14A—C14—H14B	107.635
C15—C16—C17	119.49 (10)	C5—C17—H17	108.958
C5—C17—C16	108.08 (9)	C16—C17—H17	108.950
C5—C17—C18	112.10 (11)	C18—C17—H17	108.952
C16—C17—C18	109.76 (10)	C17—C18—H18	114.8 (9)
C17—C18—C19	124.37 (12)	C19—C18—H18	120.9 (9)
C18—C19—C20	126.14 (13)	C18—C19—H19	118.2 (9)
C19—C20—C21	122.58 (12)	C20—C19—H19	115.6 (9)
C19—C20—C25	119.71 (12)	C20—C21—H21	119.284
C21—C20—C25	117.70 (12)	C22—C21—H21	119.290
C20—C21—C22	121.43 (14)	C21—C22—H22	120.210
C21—C22—C23	119.59 (16)	C23—C22—H22	120.204
C22—C23—C24	120.77 (16)	C22—C23—H23	119.611
C23—C24—C25	119.54 (15)	C24—C23—H23	119.620
O4—C25—C20	115.13 (12)	C23—C24—H24	120.229
O4—C25—C24	123.89 (13)	C25—C24—H24	120.231
C20—C25—C24	120.97 (14)	O4—C26—H26A	109.470
C6—C7—H7A	109.148	O4—C26—H26B	109.470
C6—C7—H7B	109.150	O4—C26—H26C	109.467
C8—C7—H7A	109.145	H26A—C26—H26B	109.473
C8—C7—H7B	109.144	H26A—C26—H26C	109.476
H7A—C7—H7B	107.867	H26B—C26—H26C	109.471
			
C10—O1—C11—C12	−164.47 (9)	C16—C11—C12—C13	25.24 (18)
C10—O1—C11—C16	14.26 (16)	C11—C12—C13—C14	−50.00 (15)
C11—O1—C10—C5	−13.16 (16)	C12—C13—C14—C15	49.61 (16)
C11—O1—C10—C9	165.66 (9)	C13—C14—C15—O3	160.13 (13)
C26—O4—C25—C20	−174.47 (16)	C13—C14—C15—C16	−22.03 (18)
C26—O4—C25—C24	4.6 (3)	O3—C15—C16—C11	172.68 (11)
C6—C5—C10—O1	170.15 (10)	O3—C15—C16—C17	−7.37 (17)
C6—C5—C10—C9	−8.47 (19)	C14—C15—C16—C11	−5.16 (17)
C10—C5—C6—O2	−178.59 (11)	C14—C15—C16—C17	174.80 (11)
C10—C5—C6—C7	−1.71 (17)	C11—C16—C17—C5	−21.69 (15)
C6—C5—C17—C16	−154.19 (10)	C11—C16—C17—C18	100.83 (12)
C6—C5—C17—C18	84.73 (13)	C15—C16—C17—C5	158.36 (10)
C17—C5—C6—O2	−1.49 (18)	C15—C16—C17—C18	−79.12 (13)
C17—C5—C6—C7	175.39 (9)	C5—C17—C18—C19	−129.02 (12)
C10—C5—C17—C16	22.80 (15)	C16—C17—C18—C19	110.88 (13)
C10—C5—C17—C18	−98.28 (13)	C17—C18—C19—C20	−177.35 (11)
C17—C5—C10—O1	−6.88 (18)	C18—C19—C20—C21	−26.5 (3)
C17—C5—C10—C9	174.51 (10)	C18—C19—C20—C25	154.06 (13)
O2—C6—C7—C8	−148.51 (12)	C19—C20—C21—C22	−179.46 (12)
C5—C6—C7—C8	34.64 (16)	C19—C20—C25—O4	−1.7 (2)
C6—C7—C8—C9	−57.17 (15)	C19—C20—C25—C24	179.26 (12)
C7—C8—C9—C10	46.59 (16)	C21—C20—C25—O4	178.83 (12)
C8—C9—C10—O1	166.32 (12)	C21—C20—C25—C24	−0.2 (2)
C8—C9—C10—C5	−14.9 (2)	C25—C20—C21—C22	0.0 (2)
O1—C11—C12—C13	−156.06 (10)	C20—C21—C22—C23	0.6 (3)
O1—C11—C16—C15	−175.32 (10)	C21—C22—C23—C24	−0.9 (3)
O1—C11—C16—C17	4.72 (18)	C22—C23—C24—C25	0.7 (3)
C12—C11—C16—C15	3.24 (18)	C23—C24—C25—O4	−179.08 (15)
C12—C11—C16—C17	−176.72 (11)	C23—C24—C25—C20	−0.1 (3)

Symmetry codes: (i) −*x*, *y*−1/2, −*z*+1/2; (ii) *x*−1, *y*, *z*; (iii) −*x*+1, *y*−1/2, −*z*+1/2; (iv) −*x*, −*y*+1, −*z*; (v) −*x*, *y*+1/2, −*z*+1/2; (vi) −*x*+1, *y*+1/2, −*z*+1/2; (vii) *x*+1, *y*, *z*; (viii) *x*+1, −*y*+3/2, *z*+1/2; (ix) −*x*, −*y*+1, −*z*+1; (x) *x*, −*y*+1/2, *z*+1/2; (xi) −*x*−1, −*y*+1, −*z*; (xii) −*x*−1, *y*+1/2, −*z*+1/2; (xiii) −*x*−1, −*y*+2, −*z*; (xiv) *x*, −*y*+3/2, *z*−1/2; (xv) *x*, −*y*+1/2, *z*−1/2; (xvi) −*x*−1, *y*−1/2, −*z*+1/2; (xvii) *x*, −*y*+3/2, *z*+1/2; (xviii) *x*−1, −*y*+3/2, *z*−1/2.

### Hydrogen-bond geometry (Å, °)

**Table:**

*D*—H···*A*	*D*—H	H···*A*	*D*···*A*	*D*—H···*A*
C14—H14B···O2^vii^	0.97	2.43	3.3231 (18)	153

Symmetry codes: (vii) *x*+1, *y*, *z*.
